# The antineoplastic drug, trastuzumab, dysregulates metabolism in iPSC-derived cardiomyocytes

**DOI:** 10.1186/s40169-016-0133-2

**Published:** 2017-01-18

**Authors:** Brian M. Necela, Bianca C. Axenfeld, Daniel J. Serie, Jennifer M. Kachergus, Edith A. Perez, E. Aubrey Thompson, Nadine Norton

**Affiliations:** 1 Department of Cancer Biology, Mayo Clinic, Jacksonville, FL USA; 2 Department of Health Sciences Research, Mayo Clinic, Jacksonville, FL USA; 3 Department of Hematology Oncology, Mayo Clinic, Jacksonville, FL USA

## Abstract

**Background:**

The targeted ERBB2 therapy, trastuzumab, has had a tremendous impact on management of patients with HER2+ breast cancer, leading to development and increased use of further HER2 targeted therapies. The major clinical side effect is cardiotoxicity but the mechanism is largely unknown. On the basis that gene expression is known to be altered in multiple models of heart failure, we examined differential gene expression of iPSC-derived cardiomyocytes treated at day 11 with the ERBB2 targeted monoclonal antibody, trastuzumab for 48 h and the small molecule tyrosine kinase inhibitor of EGFR and ERBB2.

**Results:**

Transcriptome sequencing was performed on four replicates from each group (48 h untreated, 48 h trastuzumab and 48 h lapatinib) and differential gene expression analyses were performed on each treatment group relative to untreated cardiomyocytes. 517 and 1358 genes were differentially expressed, p < 0.05, respectively in cardiomyocytes treated with trastuzumab and lapatinib. Gene ontology analyses revealed in cardiomyocytes treated with trastuzumab, significant down-regulation of genes involved in small molecule metabolism (p = 3.22 × 10^−9^) and cholesterol (p = 0.01) and sterol (p = 0.03) processing. We next measured glucose uptake and lactate production in iPSC-derived cardiomyocytes 13 days post-plating, treated with trastuzumab up to 96 h. We observed significantly decreased glucose uptake from the media of iPSC-derived cardiomyocytes treated with trastuzumab as early as 24 h (p = 0.001) and consistently up to 96 h (p = 0.03).

**Conclusions:**

Our study suggests dysregulation of cardiac gene expression and metabolism as key elements of ERBB2 signaling that could potentially be early biomarkers of cardiotoxicity.

**Electronic supplementary material:**

The online version of this article (doi:10.1186/s40169-016-0133-2) contains supplementary material, which is available to authorized users.

## Background

As major advances are being made to understand the biology of the disease and optimize therapeutic benefit of various treatment strategies, side effects associated with those treatments must also be understood in order to minimize the dose/treatment limiting effects of such complications. One of the most impactful therapies for breast cancer is the humanized monoclonal antibody, trastuzumab (Herceptin®), which specifically recognizes the HER2 protein encoded by the *ERBB2* gene. HER2 is part of a family of transmembrane receptors, is overexpressed in about 15–20% of invasive breast cancers, and is associated with aggressive biology and a natural history of shortened survival.

The original pivotal trial of trastuzumab in patients with HER2-positive metastatic breast cancer created concern for the cardiac safety of patients receiving HER2 inhibitors after 27% of patients treated with concurrent anthracycline (when given at a cumulative dose of >300 mg/m^2^)/cyclophosphamide/trastuzumab and 13% of those treated with trastuzumab/paclitaxel experienced some degree of cardiotoxicity [1]. Data from multiple randomized adjuvant trials and observational studies suggest that the rate of discontinuation of trastuzumab treatment ranges between 6 and 31%, mainly due to cardiotoxicity [2–10]; and in patients aged ≥65 years old, this figure increases to a notable and clinically relevant rate of 41% [11].

Trastuzumab is a monoclonal antibody that targets the protein HER2, but the mechanisms that account for trastuzumab-mediated cardiotoxicity are largely unknown. The expert consensus statement from the American Society of Echocardiography and the European Association of Cardiovascular imaging, classifies Trastuzumab as a Type II Cancer therapeutics-related cardiac dysfunction (CTRCD) agent because it does not directly cause cell damage in a cumulative dose-dependent fashion (unlike Type 1 CTCRD/anthracycline cardiotoxicity), and observed cardiac dysfunction is often reversible when treatment is discontinued [12, 13]. However classification of cardiotoxicity as Type I or II is confounded by the fact that such treatments are often given sequentially or concurrently and that echocardiography may miss subtle cardiac defects which do not change left ventricular ejection fraction (LVEF) measurement.

To some extent, these factors have been addressed in animal models, which allow single treatment with trastuzumab and more invasive testing methods. ErbB2-deficient conditional mutant adult mice were viable and displayed no overt phenotype, but physiological analysis revealed a phenotype consistent with dilated cardiomyopathy [14, 15]. Isolated cardiomyocytes from conditional mutants were more susceptible to anthracycline toxicity, demonstrating that ErbB2 signaling in cardiomyocytes is requisite for the prevention of dilated cardiomyopathy (DCM) [14].

More recent pharmacological studies in both mouse and zebrafish suggest that the link between ErbB2 inhibition and DCM is not directly linked to cardiomyocyte survival; rather that pharmacological inhibition of ErbB2/erbb2 (without combination treatment with anthracycline), can result in myofibril remodeling. Treatment of wild type mice with trastuzumab, resulted in impaired ventricular function, ultrastructural damage of heart tissue (stretched appearance and reduced thickness of ventricular cardiac myofibers), and differential expression of 15 genes involved in adaptability to cardiac contractility, hemodynamic stress, DNA repair mechanisms, apoptosis, and mitochondrial function [16]. Taken together, mouse and zebrafish studies suggest that ErbB2/*erbb2* inhibition plays a role in cardiomyocyte survival following anthracycline treatment, but also directly affects the structural organization of specific subpopulations of ventricular myofibrils, even in the absence of anthracycline.

The limitations of these studies is their reliance on genetic models of *erbb2* inhibition, or in the case of pharmacological experimentation, trastuzumab is a humanized monoclonal antibody and may not have the same efficacy in the mouse. Trastuzumab is not an option in zebrafish models, and the pharmacological inhibition in the literature [17–19] used PD168393, a compound that inhibits both EGFR and ERBB2 but is not used in the clinic.

Human induced pluripotent stem cell (hiPSC) derived cardiomyocytes are now a convenient option for the assessment cardiotoxicity of clinically relevant drugs in humans. On the basis that trastuzumab has been shown to alter the expression of genes essential for cardiac contractility in a mouse model [16] and that different gene expression profiles in multiple models of heart failure are well known [20–25], we hypothesized that detailed examination of trastuzumab induced differential gene expression in human iPSC-derived cardiomyocytes could provide insight into the genes, pathways and potential biomarkers of trastuzumab induced cardiotoxicity.

## Methods

### Compounds

Trastuzumab, was received from Genentech (South San Francisco, CA) as a gift. Lapatinib Ditosylate was purchased from LC Laboratories (Woburn, MA). We used 100 µg/mL trastuzumab to represent a clinically relevant dose. In studies of patients in which an initial dose of 4 mg/kg was followed by a weekly maintenance dose of 2 mg/kg, in combination with chemotherapy, the mean ± S.D. peak serum trastuzumab concentration at week 8 was 101.0 ± 30.6 µg/mL (n = 115). Among patients receiving trastuzumab alone, mean ± S.D. peak concentration at week 8 was 95.6 ± 35.9 µg/mL [26]. We used 2 µM lapatinib Ditosylate based on previous observations [27].

### Breast cancer cell culture

SKBR3, BT474, HCC1954, MDA-MB-453, HCC1569, and HCC1419 breast cancer cell lines were purchased from ATCC (Manassas, VA). SKBR3 and BT474 cell lines were maintained at in IMEM supplemented with 10% fetal bovine serum (FBS) and cultured at 37 °C, 5% CO_2_. HCC1954, HCC1569, and HCC1419 cells were cultured in RPMI with 10% FBS at 37 °C, 5% CO_2_. MDA-MB-453 cells were cultured in L-15 with 10% FBS at 37 °C, no CO_2_. All cells passaged with 0.25% trypsin as needed.

### Cardiomyocyte cell culture

iCell® cardiomyocytes were purchased from CELLular Dynamics International, CDI (Madison, WI) maintained according to the manufacturer’s recommended instructions. Cardiomyocytes were seeded with CDI’s plating media into fibronectin (5 µg/mL) coated wells at the appropriate density (2.4^e5^ cells per well of 12 well plate; 2^e4^ per well of 96 well plate. After 2 days, media was changed to CDI’s maintenance media and replaced every other day. Cells were maintained at 37 °C, 7% CO_2_ until 10 days after plating at which time cells were treated with the appropriate drug for 48 h and endpoint assays (RNA extraction, viability/apoptosis) performed.

### Western blot analysis

15 µg total cell lysates were prepared by briefly sonicating cell pellets in 1× lysis buffer (Cell Signaling) supplemented with a protease inhibitor mixture (Roche) and phosphatase inhibitors (PhosSTOP, Roche). Protein samples were resolved on Bolt 8% Bis–Tris Plus gels (ThermoFisher) and were electrophoretically transferred to a polyvinylidene difluoride membrane and blocked for 1 h at room temperature with 5% dry milk in TBST (50 mM Tris, 150 mM NaCl, 0.1% Tween, pH 7.5). Blots were stained with antibodies against EGFR (D38B1), HER2/ERRB2 (D8F12), HER3/ERRB3(D22C5), and HER4/ERBB4 (111B2),and GAPDH (D16H11) purchased from Cell Signaling Technology and used a dilution of 1:1000 in 5% dry milk in TBST for 1 h at 22 °C. For detection of PHLDA1 expression, blots were stained with 1;1000 dilution of anti-PHLDA1 antibody (EPR6674, abcam®). For detection of PDK4 expression, blots were stained with 2 µg/mL dilution of anti-PDK4 (NBP1-07049, Novus Biologicals). All blots were washed with three changes of TBST for a total of 45 min. Blots were then incubated in TBST buffer with 5% dry milk containing GAR-HRP (1:10,000, Cell Signaling Technology) for 1 h at room temperature. After three washes with TBST, antigen–antibody complexes were detected with the ECL Plus chemiluminescent system (Amersham Biosciences) and visualized with film.

### Transcriptome sequencing

RNA was extracted from breast cancer cell lines and iPSC-derived cardiomyocytes with the RNeasy minikit (Qiagen). RNASeq libraries were prepared with the TruSeq v2 (Illumina Inc, San Diego, Ca), and sequenced by 101 base paired-end sequencing on an Illumina HiSeq 2000 (Illumina Inc, San Diego, Ca), mRNA as per the manufacturer’s instructions. Quality control, sequence alignment and gene expression counts were obtained using MAP-R seq workflow [28]. Transcriptome data is deposited in GEO, accession number GSE91383.

### Quantitative PCR

Two-step quantitative reverse transcriptase-mediated real-time PCR (qPCR) was used to measure abundance of individual mRNAs. Equal aliquots of total RNA from samples were converted to cDNA with the High-Capacity cDNA Archive kit (Applied Biosystems), and qPCR reactions were performed in triplicate with 10 ng of cDNA and the TaqMan Universal PCR master mix (Applied Biosystems). Primer/probe sets were purchased from Applied Biosystems. Amplification data were collected with an Applied Biosystems ViiA7 detector and analyzed with ViiA7 v 1.2.4 software (Life Technologies). Data were normalized to the endogenous control POLR2A [29] and mRNA abundance was calculated using the ΔΔCT method [30].

### Metabolic assays

Glucose was measured in cell culture supernatant by Amplex® Red Glucose/Glucose Oxidase Assay (ThermoFisher). Fluorescence was measured at 530/590 nm, and glucose concentration calculated via standard curve. Lactate was measured in cell culture supernatant using the Lactate Colorimetric/Fluorometric Kit (Biovision). Fluorescence was measured at 535/587 nm.

### Apoptosis assay

Apoptosis was measured by caspase-3/7 activation with the ApoTox-Glo assay (Promega) as per the manufacturer’s instructions.

### Statistical analyses

RNASeq statistical analyses were conducted with R version 3.1.1. Transcriptome-wide differential expression analyses were undertaken via the edgeR package, version 3.8.6. Cell lines and cardiomyocytes treated with trastuzumab or lapatinib were compared to untreated samples (four replicates each group). Genes with less than two samples expressed at two counts-per-million or greater were considered beneath the threshold of detection and filtered from all analyses. To correct for multiple testing, Benjamini and Hochberg’s FDR method was applied and a threshold of q < 0.05 was considered significant.

Gene enrichment analyses were performed with the Gene Ontology (GO) Consortium database [31, 32] in each cell type following ERBB2 inhibition. For each transcriptome, we took those genes differentially expressed, p < 0.05 and searched for enrichment of gene ontology categories by biological process.

Significance of qPCR fold change, glucose uptake, lactate production and apoptosis following treatment with trastuzumab or lapatinib was analyzed by *T* test in Graphpad Prism.

## Results

### ERBB expression in iPSC-derived cardiomyocytes and cancer cell lines overexpressing ERBB2

**Fig. 1:**
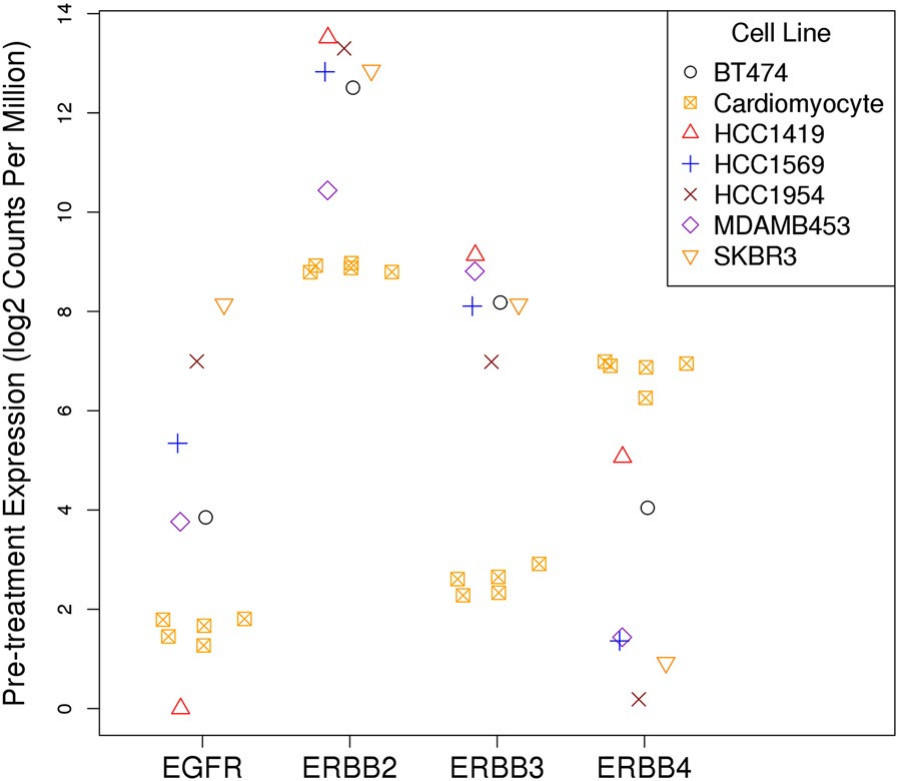
RNA expression of ERBB family in iPSC-derived cardiomyocytes and cancer cell lines overexpressing ERBB2. At the level of RNA, ERBB2-overexpressing cancer cell lines express higher levels of *ERBB2* by ~1–5 orders of magnitude relative to iPSC-derived cardiomyocytes. iPSC-derived cardiomyocytes expressed moderate levels of *ERBB4*, and low levels of *EGFR* and *ERBB3*

To put into context, ERBB2 expression levels in cancer cell lines overexpressing ERBB2 and iPSC-derived cardiomyocytes, we examined RNA and protein expression levels of the ERBB family. Gene expression of all members of the ERBB family of tyrosine kinase receptors was detected in cardiomyocytes and cancer cell lines overexpressing ERBB2 (Fig. 1). As expected, *ERBB2* expression was significantly higher in the ERBB2-overexpressing cancer cell lines (mean 12.9 log2 counts per million), but *ERBB2* was also expressed in iPSC-derived cardiomyocytes, 8.96 log2 counts per million. *ERBB2* gene expression is shown separately for each of the six cancer cell lines in Fig. 1.

**Fig. 2:**
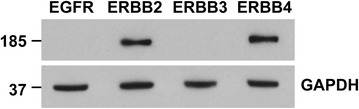
Protein expression of ERBB family in iPSC-derived cardiomyocytes. Western blot of 15 µg of total cell lysate of iPSC-derived cardiomyocytes stained for members of the ERBB family. GAPDH was used as loading control. ERBB2 and ERBB4 are expressed in iPSC-derived cardiomyocytes at the level of protein. We did not observe protein expression of EGFR and ERBB4

**Fig. 3:**
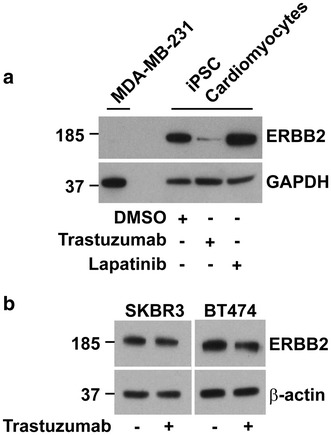
Trastuzumab down-regulates ERBB2 in iPSC-derived cardiomyocytes, but not in ERBB2 overexpressing cancer cell lines. **a** Western blot of 15 µg of total cell lysate stained for ERBB2 in MDA-MB-231 (non-ERBB2 overexpressing breast cancer cell line negative control), and iPSC-cardiomyocytes treated with trastuzumab and lapatinib. ERBB2 expression was down-regulated by trastuzumab but not by lapatinib. **b** ERBB2 expression was not altered by trastuzumab in the ERBB2 overexpressing cancer cell lines, BT474 and SKBR3

Protein levels of each ERBB family member in iPSC-derived cardiomyocytes are shown in Fig. 2. Using 15 µg of cell lysate, only ERBB2 and ERBB4 were detected at the protein level in iPSC-derived cardiomyocytes. The lack of EGFR expression in the iPSC-derived cardiomyocytes suggests that the action of lapatinib which targets EGFR and ERBB2 is not through inhibition of EGFR, but either ERBB2 or ERBB2 in combination with any off-target effects. ERBB2 protein was down-regulated by trastuzumab in iPSC-derived cardiomyocytes, but not by lapatinib. Trastuzumab did not down-regulate ERBB2 protein expression in ERBB2 overexpressing cancer cell lines (Fig. 3).

### Trastuzumab induced significant differential gene expression in iPSC-derived cardiomyocytes

517 genes were differentially expressed in iPSC-derived cardiomyocytes treated with trastuzumab at p < 0.05, of which 137 showed fold change >1.5 (49 down-regulated, 88 up-regulated). Only one gene, *IL31RA* remained significant following correction for multiple testing, fold change 3.85, p = 1.56 × 10^−6^, q = 0.025. Full differential expression results are shown in Additional file 1: Table S1.

### Overlap of trastuzumab induced differentially expressed genes is relatively small between ERBB2-overexpressing cancer cell lines and iPSC-derived cardiomyocytes

We observed a high degree of overlap in the genes expressed in iPSC-derived cardiomyocytes and ERBB2-overexpressing cancer cell lines. Of the 14,946 genes expressed above background in the cancer cell lines, 11,466 (77%) were also expressed above background in the iPSC cardiomyocytes (Additional file 2: Figure S1A). As further validation of changes in gene expression mediated by ERBB2 inhibition, we next assessed the overlap of trastuzumab induced differential gene expression between the ERBB2-overexpressing cancer cell lines and iPSC-derived cardiomyocytes. 76/11,466 (0.66%) genes expressed in both cell types were differentially expressed, p < 0.05, (Additional file 2: Figure S1B). 76 genes were differentially expressed, p < 0.05 in both cancer cell lines and iPSC-derived cardiomyocytes following treatment with trastuzumab (Table 1). 63/76 genes showed fold changes in the same direction in both cancer cell lines and iPSC-derived cardiomyocytes.

**Table 1:** 76 genes were differentially expressed in both ERBB2-overexpressing cancer cell lines and iPSC-derived cardiomyocytes, following treatment with trastuzumab, p < 0.05

Gene	ERBB2-overexpressing cancer cell lines			iPSC-derived cardiomyocytes		
	logCPM	FC	p value	logCPM	FC	p value
*DDX60*	4.37	1.31	2.32E−04	4.42	1.68	0.001
*GABRP*	3.10	1.43	0.032	0.51	2.46	0.001
*DHCR24*	9.72	0.82	0.001	5.84	0.68	0.002
*DHCR7*	7.60	0.75	1.06E−04	4.53	0.70	0.002
*BMF*	5.23	1.49	1.49E−04	6.86	1.48	0.002
*PTPRE*	2.23	1.27	0.036	3.21	1.71	0.002
*INSIG1*	6.79	0.72	9.13E−05	4.21	0.72	0.003
*TYMS*	5.54	0.81	0.030	6.02	0.67	0.003
*SCD*	10.80	0.59	0.000	6.18	0.69	0.004
*ZNF90*	1.88	0.66	0.039	2.21	0.70	0.006
*SEMA6C*	3.01	1.80	0.001	6.18	1.36	0.006
*PRSS23*	6.64	1.22	6.12E−04	4.95	1.38	0.006
*CDK1*	7.12	0.80	0.023	5.25	0.70	0.007
*REPS2*	4.96	1.15	0.021	4.96	1.46	0.007
*LDLR*	7.78	0.81	1.63E−04	5.84	0.66	0.007
*YARS*	7.38	0.89	0.027	6.97	0.79	0.007
*MSMO1*	6.95	0.80	0.002	5.51	0.72	0.008
*UBE2T*	5.57	0.87	0.023	4.74	0.71	0.012
*SEPP1*	5.87	1.32	0.006	5.56	1.29	0.015
*SREBF1*	9.23	0.85	0.008	4.06	0.69	0.015
*CLU*	8.72	1.23	9.44E−04	5.81	1.37	0.016
*TOMM40*	6.91	0.84	0.002	5.37	0.74	0.017
*ABCA1*	2.46	1.43	0.003	5.32	1.48	0.017
*NSDHL*	5.41	0.84	0.004	4.22	0.77	0.018
*KIF18B*	5.33	0.78	0.014	3.52	0.70	0.018
*ERO1LB*	4.05	1.21	0.025	3.76	1.35	0.019
*NEIL3*	4.17	0.80	0.036	1.84	0.66	0.019
*HIST2H2BE*	5.09	1.20	0.019	6.96	1.34	0.019
*TUBA1B*	7.79	0.81	0.015	7.92	0.75	0.021
*FDPS*	7.37	0.80	0.001	5.83	0.82	0.022
*ERV3*-*1*	3.53	1.18	0.027	3.95	1.38	0.023
*ACAT2*	5.76	0.72	0.002	4.80	0.74	0.024
*PPL*	6.33	1.11	0.048	4.97	1.38	0.024
*NBR1*	7.15	1.12	0.026	7.24	1.25	0.027
*PDSS1*	3.74	0.75	0.007	2.24	0.72	0.027
*CCDC28A*	3.39	1.33	6.92E−04	2.38	1.33	0.027
*ETNK1*	6.47	1.12	0.010	5.65	1.25	0.027
*SNAP23*	6.44	1.10	0.050	6.19	1.23	0.027
*TUBG1*	5.00	0.84	0.016	5.01	0.81	0.028
*RRS1*	5.59	0.85	0.004	3.69	0.72	0.029
*MATN2*	5.79	1.39	8.99E−04	5.89	1.28	0.032
*NCAPH*	5.88	0.76	0.007	3.31	0.69	0.032
*AKAP3*	0.90	1.44	0.033	1.59	1.41	0.032
*MPV17L2*	3.69	0.81	0.001	4.46	0.77	0.032
*PTGES2*	6.47	0.89	0.023	5.85	0.74	0.034
*WDR4*	4.54	0.79	0.005	2.11	0.76	0.036
*TXNIP*	7.50	1.38	5.31E−04	4.36	1.30	0.037
*TOP3A*	6.15	0.87	0.007	4.74	0.84	0.038
*PDK4*	1.08	2.20	1.80E−05	2.77	1.43	0.039
*BCAS3*	4.46	1.21	0.009	4.42	1.25	0.039
*RRM2*	8.39	0.76	0.014	5.27	0.77	0.039
*CHCHD3*	6.22	0.87	0.031	6.56	0.82	0.039
*MBNL2*	6.95	1.12	0.023	5.89	1.31	0.040
*RAB17*	5.09	1.17	0.039	2.04	1.50	0.040
*BIRC5*	6.58	0.79	0.023	4.81	0.70	0.040
*PGAM5*	6.54	0.82	3.46E−04	5.09	0.80	0.040
*IDI1*	7.37	0.82	0.003	5.76	0.82	0.043
*FADS2*	7.15	0.76	0.013	6.90	0.81	0.046
*RNASEH2A*	5.57	0.85	0.033	3.52	0.69	0.046
*NFASC*	4.95	1.55	0.005	0.68	1.58	0.048
*PAK1IP1*	5.00	0.89	0.048	2.88	0.81	0.049
*DHFR*	6.93	0.86	0.018	6.46	0.82	0.049
*FHL1*	3.30	1.49	0.041	7.24	1.21	0.050
*PRODH*	*7.11*	*1.24*	*0.005*	*2.43*	*0.59*	*0.003*
*KCNQ5*	*0.32*	*0.57*	*0.019*	*4.02*	*1.54*	*0.006*
*PIM1*	*4.28*	*1.25*	*0.005*	*3.68*	*0.72*	*0.006*
*MAP6D1*	*3.06*	*1.17*	*0.038*	*2.05*	*0.70*	*0.011*
*TLL1*	−*0.08*	*0.64*	*0.049*	*2.36*	*1.76*	*0.013*
*DYNC1LI1*	*5.67*	*0.89*	*0.022*	*7.96*	*1.28*	*0.015*
*MXRA5*	*6.22*	*0.83*	*0.019*	*1.33*	*1.90*	*0.015*
*RILPL2*	*3.16*	*1.27*	*0.012*	*2.11*	*0.72*	*0.023*
*RPL18A*	*8.66*	*1.11*	*0.028*	*5.97*	*0.77*	*0.032*
*NAGLU*	*4.69*	*1.17*	*0.023*	*3.64*	*0.76*	*0.034*
*RNF39*	*3.05*	*1.28*	*0.046*	*1.04*	*0.70*	*0.039*
*RPS18*	*9.84*	*1.11*	*0.041*	*8.29*	*0.76*	*0.040*
*ARHGAP18*	*4.99*	*1.11*	*0.040*	*0.74*	*0.67*	*0.048*

63/76 genes showed fold changes in the same direction. 13 genes with FC in the opposite direction between cancer cell lines and iPSC-derived cardiomyocytes are listed at the bottom of the table in italics. All genes are ordered by p values in cardiomyocytes

Differential gene expression analyses were performed in six cancer cell lines overexpressing ERBB2 (BT474, HCC1419, HCC1569, HCC1954, MDAMB453 and SKBR3) and in iPSC-derived cardiomyocytes following treatment with trastuzumab

*iPSC* induced pluripotent stem cell, *logCPM* log 2 counts per million, *FC* fold change

### Validation of trastuzumab induced differentially expressed genes in iPSC-derived cardiomyocytes by additional ERBB2 targeted compounds

We also performed transcriptome sequencing on iPSC-derived cardiomyocytes following 48 h treatment with the small molecule tyrosine kinase inhibitor, lapatinib, and examined differential gene expression of treated versus untreated cells. We observed a total of 1358 genes that were differentially expressed following treatment with lapatinib at p < 0.05, of which 533 showed fold change >1.5 (271 up-regulated and 262 down-regulated). 75 genes remained significant q < 0.05 after correction for multiple testing, the most significant of which was *PHLDA1*, p = 8.89 × 10^−9^, q = 0.0001, fold change 0.29. Differential gene expression data for all genes following treatment with lapatinib is shown in Additional file 3: Table S2.

38/137 (28%) of genes that were differentially expressed in iPSC cardiomyocytes following treatment with trastuzumab, were also differentially expressed following treatment with lapatinib, p < 0.05 and fold change >1.5, (Table 2), including the most significantly differentially expressed genes from both drugs, *IL31RA* from trastuzumab and *PHLDA1* from lapatinib. 93% of genes that were differentially expressed following treatment with lapatinib were not significantly changed by treatment with trastuzumab, demonstrating the wider range of effect of the tyrosine kinase inhibitor relative to monoclonal antibody.

**Table 2:** Differentially expressed genes in cardiomyocytes following ERBB2 inhibition

Gene	logCPM	iPSC cardiomyocytes treated with trastuzumab		iPSC cardiomyoytes treated with lapatinib	
		FC	p value	FC	p value
PHLDA1	4.366	0.53	0.003	0.29	8.89E−09
SLC6A6	3.051	0.53	0.006	0.27	2.35E−08
RGS4	1.850	0.66	0.022	0.41	1.15E−06
LDLR	5.843	0.66	0.007	0.48	1.98E−06
RP11-290L1.3	2.017	0.44	0.004	0.26	3.09E−06
FSTL5	1.461	2.26	2.25576E−05	2.23	2.93E−05
EGR1	3.271	0.40	6.21035E−05	0.39	4.71E−05
KRT6A	−1.435	0.00	1.51E−04	0.00	1.42E−04
STK32A	1.068	1.97	6.48E−04	2.08	2.05E−04
PRRT4	2.336	1.72	2.62E−04	1.68	4.71E−04
FAM71F2	0.724	1.91	0.007	2.22	0.001
ZNF483	1.654	1.76	0.018	2.22	0.001
ACRC	0.475	1.65	0.025	2.09	0.001
RP11-285F7.2	0.797	1.66	0.020	2.03	0.001
LINC00842	3.506	1.64	0.001	1.61	0.002
RP3-434O14.8	0.954	2.14	0.001	1.97	0.002
NFASC	0.677	1.58	0.048	1.99	0.002
PMCH	0.823	0.64	0.047	0.51	0.003
CAV3	0.797	1.58	0.027	1.81	0.004
AL132989.1	0.577	2.04	0.012	2.18	0.006
SDC1	1.833	0.62	0.013	0.60	0.007
SLC28A1	1.089	1.96	0.028	2.25	0.008
KIAA1107	2.568	1.54	0.028	1.68	0.008
PLK3	0.683	0.55	0.006	0.56	0.009
SLC7A7	1.628	2.00	1.39564E−05	1.51	0.011
GRM8	0.678	1.92	0.011	1.89	0.012
NPY4R	0.487	1.85	0.032	2.06	0.012
FAM110A	1.255	0.64	0.011	0.66	0.016
GFAP	1.856	0.62	0.006	0.66	0.017
APBB1IP	0.323	2.46	0.003	2.06	0.019
IL31RA	0.956	3.85	1.56156E−06	1.94	0.020
RPP25	2.028	0.65	0.045	0.61	0.020
AC007036.5	2.769	1.64	0.023	1.66	0.020
LINC00327	0.750	1.59	0.009	1.51	0.021
PKN3	2.451	0.65	0.024	0.66	0.024
MTFP1	2.980	0.64	0.037	0.62	0.026
PROM1	1.535	1.69	0.010	1.55	0.031
ATF4P3	1.473	0.58	0.007	0.66	0.038

Overlap of trastuzumab and lapatinib differentially expressed genes in cardiomyocytes, fold change >1.5, p < 0.05

*iPSC* induced pluripotent stem cell, *FC* fold change

**Fig. 4:**
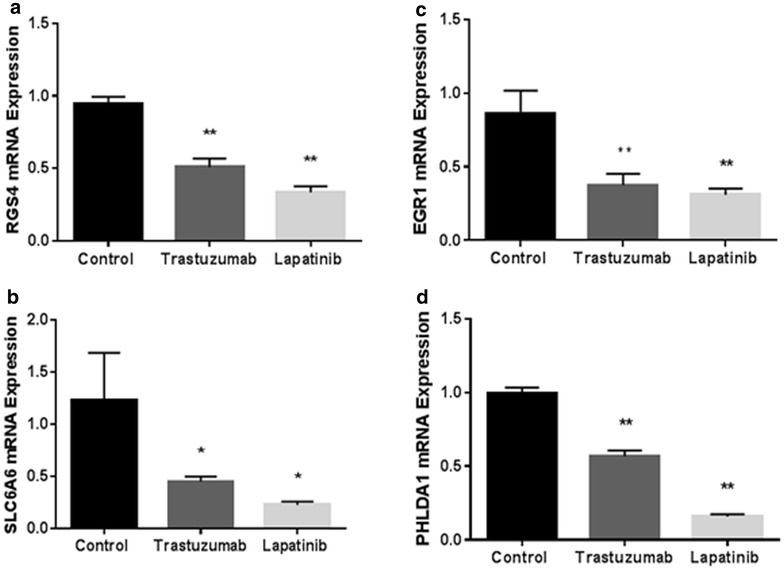
qPCR validation of differential expression of *RGS4, EGR1, SLC6A6* and *PHLDA1* by trastuzumab and lapatinib in iPSC-derived cardiomyocytes. Standard deviation (SD) calculated from three replicates. Down-regulation of: **a** *RGS4,* **b** *SLC6A6,* **c** *EGR1* and **d** *PHLDA1* by trastuzumab and lapatinib was confirmed by qPCR. Student’s T-test, unequal values, p < 0.05*; p < 0.01**

**Fig. 5:**
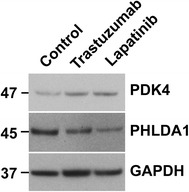
Trastuzumab and lapatinib induced down-regulation of PHLDA1 protein expression and up-regulation of PDK4 protein expression. Western blot of 15 μg of total cell lysate of iPSC-derived cardiomyocytes stained for PHLDA1 and PDK4

To validate the differentially expressed genes identified by transcriptome sequencing, we selected genes with fold changes >1.5, p < 0.05, following treatment with both trastuzumab and lapatinib: *EGR1*, *PHLDA1*, *RGS4*, *SLC6A6* for qPCR in RNA extracted from two separate batches of iPSC-derived cardiomyocytes (Fig. 4). To further evaluate ERBB2 inhibition of these genes, we also treated cells from a new batch of iPSC-derived cardiomyocytes with additional ERBB2 inhibitors, neratinib, afatinib and poziotinib. *PHLDA1* and *SLC6A6* were significantly down-regulated by all inhibitors (Additional file 4: Figure S2). We also confirmed down-regulation of PHLDA1 at the level of protein expression by Western blot, (Fig. 5).

### ERBB2 inhibition down-regulates genes involved in metabolism in iPSC-derived cardiomyocytes and ERBB2-overexpressing cancer cell lines

We used gene ontology (GO) [31, 32] to perform gene enrichment analyses in each cell type following ERBB2 inhibition. For each transcriptome, we took those genes differentially expressed at p < 0.05 and searched for enrichment of gene ontology categories by biological process. Following Bonferroni correction of each GO term, a total of 272 GO terms were associated at p < 0.05 with differentially expressed genes in ERBB2-overexpressing cancer cell lines treated with trastuzumab. Not surprisingly, the majority of these terms related to cell cycle processes, with mitotic cell cycle, GO:0000278 being the most significant, p = 2.62 × 10^−42^ (Additional file 5: Table S3). We observed a similar trend in cardiomyocytes treated with lapatinib. 88 GO terms were associated at p < 0.05 with differentially expressed genes, the most significant terms being, mitotic nuclear division, GO:0007067, p = 1.09 × 10^−15^ and mitotic cell cycle process, GO:1903047, p = 1.68 × 10^−14^ (Additional file 6: Table S4).

However, in cardiomyocytes treated with trastuzumab, following Bonferroni correction, only 20 GO terms were associated at p < 0.05 with the differentially expressed genes, all of which related to metabolism. The most significant GO term was small molecule metabolic process, p = 3.22 × 10^−9^ (which included the taurine transporter, *SLC6A6*, already shown to be significantly down-regulated by multiple ERBB2 inhibitors in qPCR validation) and multiple terms relating to cholesterol processing were associated [cholesterol biosynthetic process (GO:0006695), cholesterol metabolic process (GO:0008203) and sterol biosynthetic process (GO:0016126) (Additional file 7: Table S5)]. Post-hoc examination of the differentially expressed genes within these categories identified the same set of recurring genes (*LDLR*, *SREBF1*, *ABCA1*, *CLN6*, *MSMO1*, *INSIG1*, *NSDHL*, *DHCR24*, *DHCR7*, *IDI2*, *FDPS*, *IDI1*) detailed in Additional file 8: Table S6, 11/12 of which were down-regulated by trastuzumab.

The same GO terms relating to small molecule metabolic process and cholesterol metabolic process in cardiomyocytes treated with trastuzumab, were also significant in cardiomyocytes treated with lapatinib (GO:0044281, p = 4.94 × 10^−4^; GO:0008203, p = 3.90 × 10^−3^ respectively) and ERBB2-overexpressing cells treated with trastuzumab (GO:0044281, p = 1.23 × 10^−10^; GO:0008203, p = 1.35 × 10^−3^) (Table 3).

**Table 3:** Gene ontology enrichment analysis

	Small molecule metabolic process (GO:0044281)	Cholesterol metabolic process (GO:0008203)	Sterol metabolic process (GO:0016125)	Cholesterol biosynthetic process (GO:0006695)	Sterol biosynthetic process (GO:0016126)
iPSC cardiomycytes treated with trastuzumab					
Genes uploaded to enrichment analysis	428/517	428/517	428/517	428/517	428/517
Uploaded genes in category	98	12	NS	8	8
Expected genes in category	45.92	2.24		0.78	0.88
Category fold enrichment	2.13	>5		>5	>5
p value (adjusted)	3.22E−09	0.030		0.013	0.031
iPSC cardiomycoytes treated with lapatinib					
Genes uploaded to enrichment analysis	1077/1358	1077/1358	1077/1358	1077/1358	1077/1358
Uploaded genes in category	173	21	21	12	12
Expected genes in category	115.54	5.64	6.31	1.97	2.22
Category fold enrichment	1.5	3.72	3.33	>5	>5
p value (adjusted)	4.94E−04	0.004	0.022	0.009	0.030
ERBB2-overexpressing cancer cell lines treated with trastuzumab					
Genes uploaded to enrichment analysis	2097/2272	2097/2272	2097/2272	2097/2272	2097/2272
Uploaded genes in category	340	32	32	22	22
Expected genes in category	224.97	10.98	12.29	3.83	4.33
Category fold enrichment	1.51	2.91	2.6	>5	>5
p value (adjusted)	1.23E−10	0.001	0.014	1.12E−06	1.06E−05

Differentially expressed genes following treatment with trastuzumab or lapatinib were assessed for enrichment in gene ontology categories of biological process. 517 genes were significantly differentially expressed in iPSC-derived cardiomyocytes following treatment with trastuzumab. 428/517 genes were recognized within the Gene Ontology (GO) Consortium reference dataset of 20,814 genes and analyzed for enrichment. Similarly, of 1358 differentially expressed genes following treatment of iPSC-derived cardiomyocytes with lapatinib, 1077 were recognized in the GO reference set and analyzed for enrichment. Of 2272 differentially expressed genes following treatment with trastuzumab in ERBB2-overexpressing cancer cell lines, 2097 were recognized in the GO reference set and analyzed for enrichment. Enrichment analyses showed 1.5- >5-fold enrichment of genes in cholesterol processing pathways relative to expected, based on the annotated reference set of 20,814 genes

### Trastuzumab induced ERBB2 inhibition down-regulates glucose uptake in iPSC-derived cardiomyocytes

**Fig. 6:**
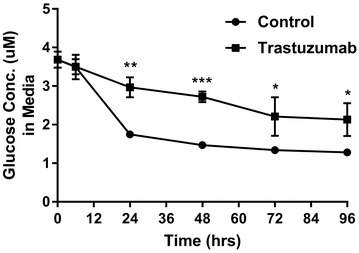
Down-regulation of glucose levels in the media of iPSC-derived cardiomyocytes following treatment with trastuzumab. Standard deviation (SD) calculated from three replicates. Time course of glucose concentration in the media of iPSC-derived cardiomyocytes treated with trastuzumab versus untreated (control). *p = 0.04 (72 h), p = 0.026 (96 h); **p = 0.001 (24 h), ***p < 0.0002 (48 h)

Following the link to trastuzumab induced differential expression of genes involved in metabolism, which included up-regulation (confirmed at the level of protein, Fig. 5), of PDK4, an inhibitor of the pyruvate dehydrogenase complex and hence glycolysis, and down-regulation of multiple genes located in the mitochondria, perhaps reflecting a reduction in oxidative phosphorylation, (*ALDH1L2*, *SLC27A3*, *SLC25A25*, *SLC25A5*, *SLC25A6* and *UCP2,* fold change and p values in Additional file 1: Table S1), we next examined the effect of trastuzumab on glycolysis. Following treatment with trastuzumab, we measured glucose uptake (based on measuring the amount of glucose in the media) up to 96 h. Cardiomyocytes treated with trastuzumab showed significantly higher amounts of glucose in the media (hence lower glucose uptake) as early as 24 h post-treatment (p = 0.001), and this difference remained significant up to 96 h post-treatment (p = 0.03), (Fig. 6).

### Trastuzumab induced ERBB2 inhibition did not alter lactate production in iPSC-derived cardiomyocytes

**Fig. 7:**
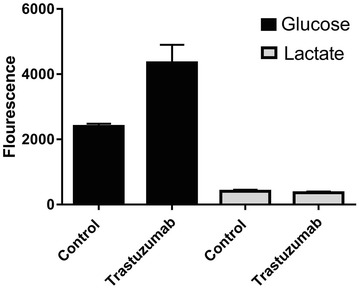
Glucose and lactate levels in the media of iPSC-derived cardiomyocytes following 48 h treatment with trastuzumab. Standard deviation (SD) calculated from three replicates. Glucose levels were significantly higher in the media of iPSC-derived cardiomyocytes treated with trastuzumab relative to untreated cells, *p = 0.004. Levels of lactate were not significantly different between iPSC-derived cardiomyocytes treated with trastuzumab and untreated cell, p = 0.329

Pyruvate can be converted to either lactate in the glycolytic pathway, or to acetyl co-enzyme A via the pyruvate dehydrogenase complex, linking glycolysis to the tricarboxylic (TCA) cycle and fatty acid synthesis. Our differential expression data showed up-regulation of *PDK4*, an inhibitor of the pyruvate dehydrogenase complex, suggesting that trastuzumab could increase lactate production. We measured lactate production in cardiomyocyte media following 48 h treatment with trastuzumab. However, we did not observe any change in lactate production, despite significantly lower glucose uptake (Fig. 7).

## Discussion

Current standard of care requires patients to receive trastuzumab for 12 months during which time they are monitored by echocardiography. Those patients experiencing significant declines in LVEF are treated with standard heart failure medications such as beta blockers or ACE inhibitors, and if LVEF does not recover, trastuzumab may be withheld. We hypothesized that identifying early changes in gene expression mediated by trastuzumab could potentially identify novel therapeutic targets for prevention of cardiac side-effects or potential biomarkers of drug-induced cardiotoxicity. We used commercially available, human iPSC-derived cardiomyocytes from a “healthy” female individual, treated with clinically relevant ERBB2 targeted therapies, trastuzumab and lapatinib to identify early therapy induced changes. We identified two groups of genes that may be relevant in the management of cardiotoxicity.

Firstly, the small group of significantly differentially expressed genes in iPSC-derived cardiomyocytes treated with trastuzumab and lapatinib, related to cardiac dysfunction and ischemic injury. *EGR1* and *PHLDA1*, both down-regulated in our experiments, are significantly upregulated in ischemic preconditioning [17], suggesting perhaps that prevention of this change in gene expression could be protective against ERBB2 therapy related cardiotoxicity. Absence of regulator of G-protein signaling 4 (RGS4) predisposes to atrial fibrillation and is associated with abnormal calcium handling [33] and activation of RGS4 mediates the cardioprotective effects of natriuretic peptides in the heart [34], of which natriuretic peptide A, *NPPA*, was also significantly down-regulated by trastuzumab and lapatinib. Solute carrier family 6, member 6 (*SLC6A6*), is a transporter of taurine, an endogenous sulfur-containing beta-amino acid, associated with calcium handling, protection against ischemia–reperfusion injury, heart failure ischemic heart disease, and diabetic cardiomyopathy [35]. *Slc6a6* knock out mouse models demonstrate a phenotype of dilated cardiomyopathy with cardiac atrophy [36], lower fasting blood glucose, acceleration of glycolysis in skeletal muscle and susceptibility to development of obesity when placed on a high fat diet [37]. Taken together, our findings of trastuzumab induced down-regulation of *SLC6A6* in human iPSC-derived cardiomyocytes, and the reported phenotypes of dilated cardiomyopathy and altered metabolism in Slc6a6 knock-out mice provide a potential mechanism towards trastuzumab related cardiotoxicity.

The second group of trastuzumab regulated genes in our iPSC-derived cardiomyocyte model involved metabolism. Enrichment analyses identified gene ontology categories: small molecule metabolism, cholesterol and sterol metabolism and cholesterol and sterol biosynthesis, and expression of the majority of genes in these categories was down-regulated across iPSC-derived cardiomyocytes treated with trastuzumab and lapatinib and also in cancer cell lines overexpressing ERBB2. In the case of iPSC-derived cardiomyocytes treated with trastuzumab, specifically, only metabolic gene ontology categories were significantly enriched. This is in contrast to ERBB2-overexpressing cancer cell lines treated with trastuzumab, in which the most significantly enriched categories related to cell cycle. We note at this point, a similar pattern in trastuzumab-induced down-regulation of ERBB2 protein levels between cancer cells and cardiomyocytes. In our data, and that of others, ERBB2 was down-regulated by trastuzumab in cardiomyocytes [38] but not in ERBB2 overexpressing cancer cell lines [39]. This is not surprising given the different mechanisms of inhibition of ERBB2 signaling and trafficking between cancer cells and cardiomyocytes [40, 41], in which treatment results in reduced proliferation and induction of apoptosis in cancer cell lines [42, 43] and reduced contractility without apoptosis in cardiomyocytes, (Additional file 9: Figure S3) [12, 44].

Regards enrichment of differential expression of metabolism related genes, the largest metabolic category, was small molecule metabolism (N = 98 genes), which included the taurine transporter, *SLC6A6*, key enzymes in glucose metabolism (*PDK4* and *GLA*) and mitochondrial transporters (*SLC27A3*, *SLC25A25*, *SLC25A5*, *SLC25A6*). Due to the high energy demand of the heart, cardiac metabolism is highly flexible. In the ‘normal/healthy’ heart, mitochondria are primarily fueled by the metabolites of fatty acids and carbohydrates, but under chronic pathological conditions, the heart has the capacity to remodel metabolic pathways, and subsequently, myocardial energetics and contractile function [45], although these data relate mostly to reprogramming in response to pathological hypertrophy, characterized by increased reliance on glucose metabolism and decreased fatty acid oxidation. The effect of trastuzumab, on glucose uptake in iPSC-derived cardiomyocytes suggests a different metabolic change to that observed in cardiac hypertrophy. We observed significantly more glucose in the media of iPSC-derived cardiomyocytes treated with trastuzumab within 24 h and constantly up to 96 h, indicative of a lower uptake of glucose as a result of trastuzumab, with negligible change in lactate. These observations are in line with studies of “lipid status” responsiveness, whereby up-regulation of PDK4, facilitates fatty acid oxidation by “sparing” pyruvate for oxaloacetate formation [46, 47]. In heart and skeletal muscle, increased anaplerotic entry of pyruvate into the TCA cycle as oxaloacetate facilitates entry of acetyl co-A derived from fatty acid β-oxidation into the TCA cycle through increased citrate formation, which in turn, acts as a sensor of fatty acid abundance and suppresses glucose uptake [46].

Observations of trastuzumab induced changes in metabolism are clinically relevant in light of potential metabolic therapies for heart failure. For example, dichloracetate (DCA), a drug shown to improve cardiac function in right ventricular hypertrophy and failure [48, 49], increases pyruvate dehydrogenase activity by inhibiting PDK, and perhaps could potentially reverse the effects of trastuzumab. The efficacy of DCA treatment has also been demonstrated in functional recovery during reperfusion in multiple animal models [50–53]. Lewandowski and White [52] demonstrated that counteracting depressed pyruvate oxidation enhanced contractile recovery in post-ischemic rabbit hearts. Of note, two of the most consistently down-regulated genes following ERBB2 inhibition, were *EGR1* and *PHLDA1*, which have also been shown to be up-regulated in models of ischemic preconditioning [17].

In summary our transcriptome based approach of trastuzumab induced changes in iPSC-derived cardiomyocytes identified significant down-regulation of expression of genes associated with ischemic preconditioning and cardiac dysfunction, and more significantly with genes involved in metabolism. The main caveats of the study are the reliance on an in vitro model of iPSC-derived cardiomyocytes which may not reflect fully mature differentiated human cardiomyocytes and does not address potential immune response, and lack of functional data. However, our reported changes of trastuzumab mediated differential gene expression in cardiomyocytes were validated with additional ERBB2 targeted compounds and we also observed considerable overlap in ERBB2-overexpressing cancer cell lines which we interpret as strong evidence that ERBB2 inhibition is responsible for our reported changes in gene expression. We acknowledge, it is not proven that these specific genes functionally contribute to dysregulation of glycolysis in iPSC-derived cardiomyocytes, but we note that knock out of *slc6a6*, one of the most consistently, differentially expressed genes in our human study, resulted in a phenotype of accelerated glycolysis in skeletal muscle in a mouse model [36].

## Additional files

**Additional file 1: Table S1.** RNASeq differential expression analysis of iPSC derived cardiomyocytes following 48 h treatment with trastuzumab.

**Additional file 2: Figure S1.** Overlap of genes expressed above background (A) and differentially expressed genes (B) in iPSC-derived cardiomyocytes and ERBB2-overexpressing cancer cells.

**Additional file 3: Table S2.** RNASeq differential expression analysis of iPSC derived cardiomyocytes following 48 h treatment with lapatinib.

**Additional file 4: Figure S2.** Differential expression in iPSC-derived cardiomyocytes following treatment withadditional tyrosine kinase inhibitors of ERBB2 (afatinib, lapatinib, neratinib, poziotinib). Standard deviation (SD) calculated from three replicates. (A) RGS4, (B) SLC6A6, (C) EGR1 and (D) PHLDA1. Student’s T-test, unequal values, p < 0.05*; p < 0.01**.

**Additional file 5: Table S3.** Gene ontology enrichment analysis of trastuzumab induced differentially expressed genes in ERBB2 overexpressing cancer cell lines.

**Additional file 6: Table S4.** Gene ontology enrichment analysis of lapatinib induced differentially expressed genes in iPSC dervied cardiomyocytes.

**Additional file 7: Table S5.** Gene ontology enrichment analysis of trastuzumab induced differentially expressed genes in iPSC dervied cardiomyocytes.

**Additional file 8: Table S6.** Differentially expressed genes in Gene ontology categories of cholesterol and sterol metabolism and biosynthesis.

**Additional file 9: Figure S3.** Treatment of iPSC-derived cardiomyocytes with trastuzumab did not result in apoptosis. Standard error (SD) calculated from four replicate reactions.
