# Supplementary figures and images for: The antineoplastic drug, trastuzumab, dysregulates metabolism in iPSC-derived cardiomyocytes
Source: Clin Transl Med. 2017 Jan 18;6:5. doi: 10.1186/s40169-016-0133-2 (PMC5243239; doi:10.1186/s40169-016-0133-2)

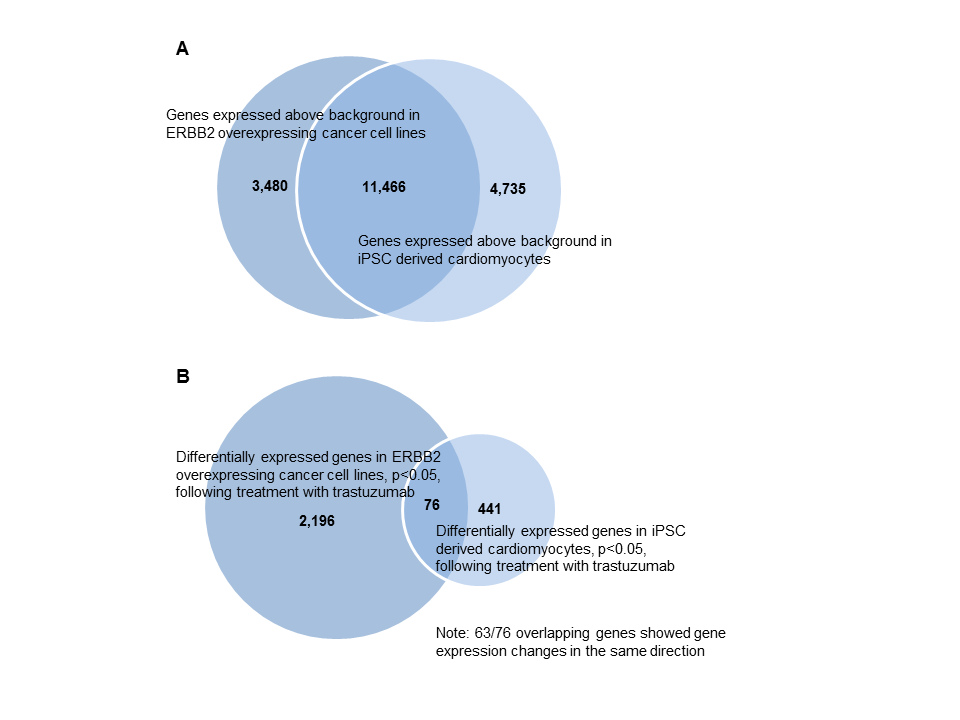

Supplement: Supplementary file 2 — Additional file 2: Figure S1. Overlap of genes expressed above background (A) and differentially expressed genes (B) in iPSC-derived cardiomyocytes and ERBB2-overexpressing cancer cells. [file 40169_2016_133_MOESM2_ESM.tif]

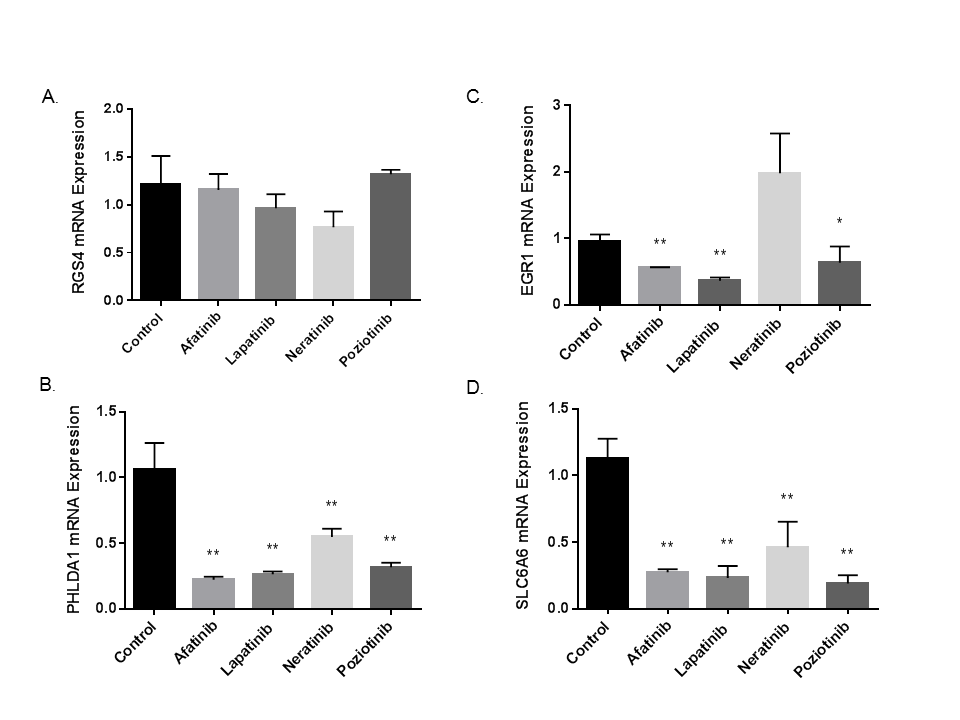

Supplement: Supplementary file 4 — Additional file 4: Figure S2. Differential expression in iPSC-derived cardiomyocytes following treatment withadditional tyrosine kinase inhibitors of ERBB2 (afatinib, lapatinib, neratinib, poziotinib). Standard deviation (SD) calculated from three replicates. (A) RGS4, (B) SLC6A6, (C) EGR1 and (D) PHLDA1. Student’s T-test, unequal values, p < 0.05*; p < 0.01**. [file 40169_2016_133_MOESM4_ESM.tif]
